# Supplementary material for: DMRforPairs: identifying Differentially Methylated Regions between unique samples using array based methylation profiles
Source: BMC Bioinformatics. 2014 May 15;15:141. doi: 10.1186/1471-2105-15-141 (PMC4046028; doi:10.1186/1471-2105-15-141)
Supplement: Additional file 2 — DMRforPairs output for the comparison of A431-MCF7 and NA17018-NA17105. Please start from the HTML files in each folder. Available via the BMC Bioinformatics website. [file 1471-2105-15-141-S2.zip › 1394847754114233_MOESM2_ESM/A431_MCF7/figures/23744.pdf]

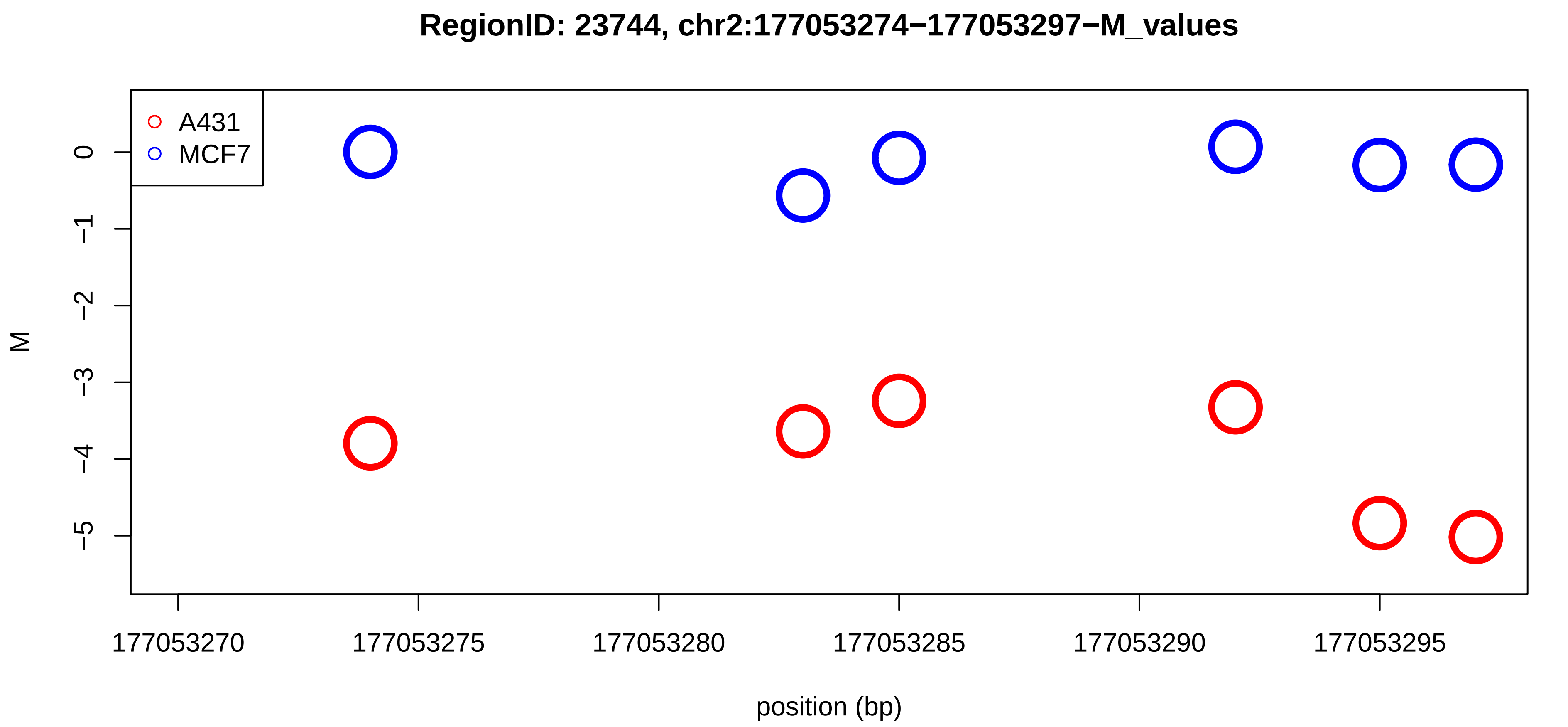

RegionID: 23744, chr2:177053274−177053297−M_values
A431
MCF7
M
0
−1
−2
−3
−4
−5
177053270
177053275
177053280
177053285
177053290
177053295
position (bp)


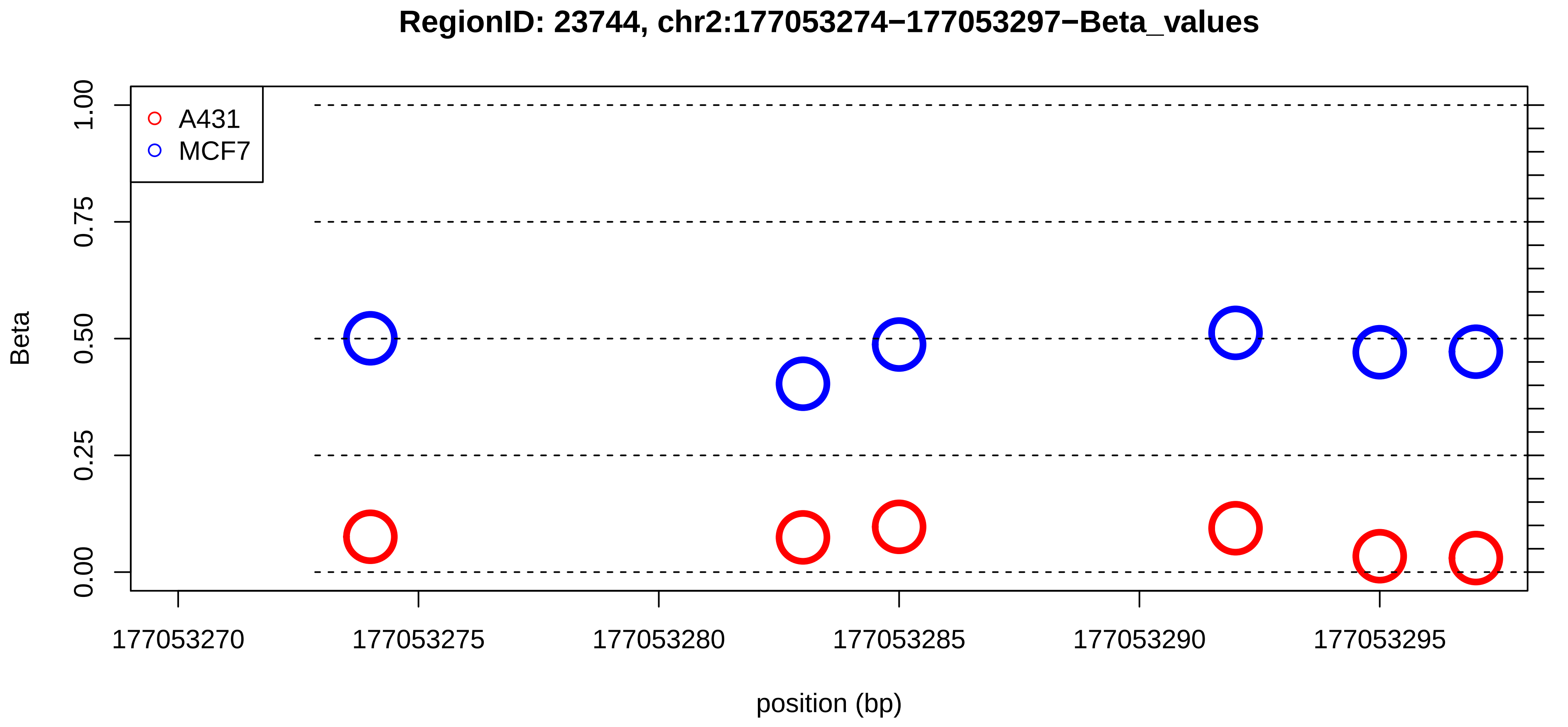

RegionID: 23744, chr2:177053274−177053297−Beta_values
A431
MCF7
Beta
1.00
0.75
0.50
0.25
0.00
177053270
177053275
177053280
177053285
177053290
177053295
position (bp)

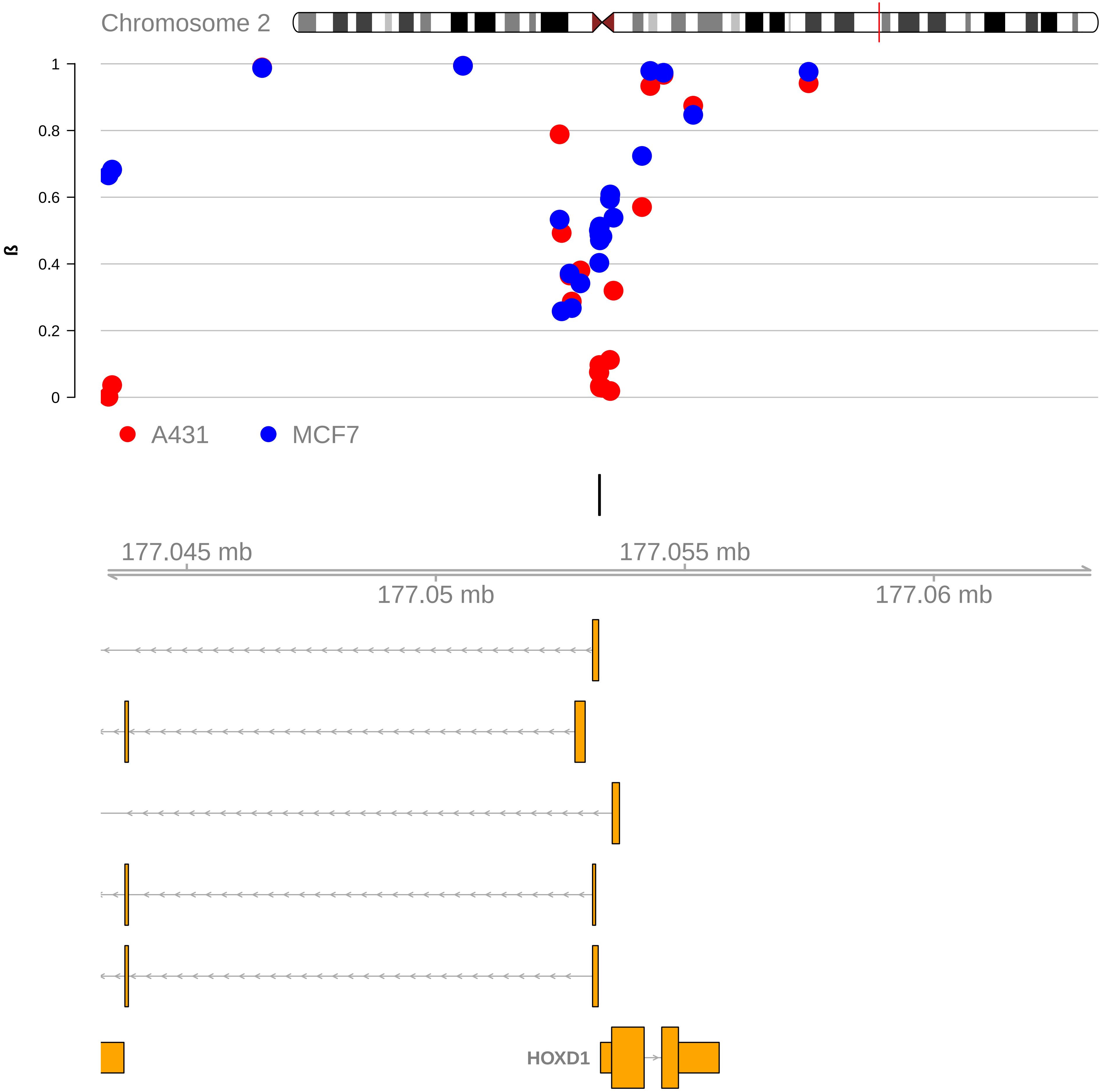

Chromosome 2
1
0.8
0.6
0.4
0.2
0
ß
A431
MCF7
177.045 mb
177.055 mb
177.05 mb
177.06 mb
HOXD1
